# Economic analysis of veterans affairs initiative to prevent methicillin-resistant *Staphylococcus aureus* infections

**DOI:** 10.1186/2047-2994-4-S1-O56

**Published:** 2015-06-16

**Authors:** RE Nelson, MH Samore, K Khader, M Jones, VW Stevens, ME Evans, ML Schweizer, E Perencevich, MA Rubin

**Affiliations:** 1 VA Salt Lake City Healthcare System, Salt Lake City, UT, USA; 2 University of Utah, Salt Lake City, UT, USA; 3 Veterans Affairs Medical Center, Lexington, KY, USA; 4 University of Kentucky, Lexington, KY, USA; 5 MRSA/MDRO Program, National Infectious Diseases Service, Veterans Health Administration, Iowa City, IA, USA; 6 University of Iowa, Iowa City, IA, USA; 7 Iowa City Veterans Affairs Health Care System, Iowa City, IA, USA

## Introduction

Methicillin resistant *Staphylococcus aureus* (MRSA) is a common cause of healthcare-associated infections (HAIs) in the US. In October 2007, the Department of Veterans Affairs (VA) launched the National MRSA Prevention Initiative, a nationwide effort to reduce MRSA transmission through universal screening and isolation.

## Objectives

The objective of this analysis was to evaluate the cost-effectiveness and the budget impact of the initiative.

## Methods

We developed an economic model using published data on the rate of MRSA HAIs in the VA from October 2007 to September 2010, recently generated estimates of the costs of MRSA HAIs, and the costs associated with the intervention obtained through a microcosting approach. To estimate the rate of MRSA HAIs that would have occurred if the initiative had not been implemented, we used the baseline rate of MRSA HAIs at the beginning of the initiative and two different assumptions of the rate of change: (1) no change and (2) a downward temporal trend in MRSA HAIs rates observed in other healthcare systems in the US during the same time frame. Effectiveness was measured in life-years (LYs) gained. This analysis did not incorporate changes in HAIs due to other organisms, which also may have been affected by this initiative.

## Results

We found that during fiscal years 2008-2010, the initiative resulted in an estimated 2,102-3,870 fewer MRSA HAIs. The initiative itself was estimated to cost $93 million over this 3-year period while the cost savings from prevented MRSA HAIs ranged from $28-73 million. The incremental cost-effectiveness of the initiative ranged from $1,648-$8,666/LY. The overall impact on the VA’s budget ranged from $20-$55 million.

## Conclusion

A national MRSA surveillance and prevention strategy in VA may have prevented a substantial number of MRSA HAIs. The savings associated with the prevented infections helped to offset some but not all of the cost of the initiative.

## Disclosure of interest

None declared.

